# Effects of DNA Damage and Oxidative Stress in Human Bronchial Epithelial Cells Exposed to PM_2.5_ from Beijing, China, in Winter

**DOI:** 10.3390/ijerph17134874

**Published:** 2020-07-06

**Authors:** Bing-Yu Niu, Wen-Ke Li, Jiang-Shuai Li, Qi-Hao Hong, Sara Khodahemmati, Jing-Feng Gao, Zhi-Xiang Zhou

**Affiliations:** 1 College of Life Science and Bioengineering, Beijing University of Technology, Beijing 100124, China; tangduo@emails.bjut.edu.cn (B.-Y.N.); liwk2018@emails.bjut.edu.cn (W.-K.L.); lijiangshuai@emails.bjut.edu.cn (J.-S.L.); hongqihao@emails.bjut.edu.cn (Q.-H.H.); 2 National Engineering Laboratory for Advanced Municipal Wastewater Treatment and Reuse Technology, Beijing University of Technology, Beijing 100124, China; SARA.KHODAHEMMATI@emails.bjut.edu.cn (S.K.); gao.jingfeng@bjut.edu.cn (J.-F.G.)

**Keywords:** PM_2.5_, DNA damage, oxidative stress, DNA repair gene, human bronchial epithelial cells

## Abstract

Epidemiological studies have corroborated that respiratory diseases, including lung cancer, are related to fine particulate matter (<2.5 μm) (PM_2.5_) exposure. The toxic responses of PM_2.5_ are greatly influenced by the source of PM_2.5_. However, the effects of PM_2.5_ from Beijing on bronchial genotoxicity are scarce. In the present study, PM_2.5_ from Beijing was sampled and applied in vitro to investigate its genotoxicity and the mechanisms behind it. Human bronchial epithelial cells 16HBE were used as a model for exposure. Low (67.5 μg/mL), medium (116.9 μg/mL), and high (202.5 μg/mL) doses of PM_2.5_ were used for cell exposure. After PM_2.5_ exposure, cell viability, oxidative stress markers, DNA (deoxyribonucleic acid) strand breaks, 8-OH-dG levels, micronuclei formation, and DNA repair gene expression were measured. The results showed that PM_2.5_ significantly induced cytotoxicity in 16HBE. Moreover, the levels of reactive oxygen species (ROS), malondialdehyde (MDA), and cellular heme oxygenase (HO-1) were increased, and the level of glutathione (GSH) was decreased, which represented the occurrence of severe oxidative stress in 16HBE. The micronucleus rate was elevated, and DNA damage occurred as indicators of the comet assay, γ-H2AX and 8-OH-dG, were markedly enhanced by PM_2.5_, accompanied by the influence of 8-oxoguanine DNA glycosylase (OGG1), X-ray repair cross-complementing gene 1 (XRCC1), and poly (ADP-ribose) polymerase-1 (PARP1) expression. These results support the significant role of PM_2.5_ genotoxicity in 16HBE cells, which may occur through the combined effect on oxidative stress and the influence of DNA repair genes.

## 1. Introduction

In recent years, air pollution particulate matter (PM), especially that with an aerodynamic diameter <2.5 μm (PM_2.5_), has been shown to be an important factor influencing human health risk. Due to theirs size, fine particles and ultra fine particles enter the body through breathing and deposits deeply in the terminal bronchioles and alveoli where it is eventually internalized across the air–blood barrier and then translocated to the endothelium and to the blood circulation, triggering a wide range of diseases [1]. Gualtieri et al. used air liquid interface cultured bronchial epithelial cells, for the first time, they obtained the model for lung deposition by a direct exposure of PM under environmental condition. The results show that for the tracheobronchial and alveolar regions of the respiratory system, ultra fine particles represent 83% and 92% of these particles [2]. PM_2.5_ exposure has been directly associated with many respiratory diseases, including asthma, chronic obstructive pulmonary disease, pneumonia, and even lung cancer [3]. According to the International Agency for Research on Cancer (IARC) in 2013, PM_2.5_ in outdoor air pollution had been classified individually as a new carcinogen to humans [4]. Data from epidemiological studies have consistently disclosed a relationship between lung cancer and PM_2.5_ exposure, especially in China, where air pollution is the most serious in the world [5].

Numerous studies have documented that PM_2.5_ exposure can cause severe genotoxic effects in animals and cells [5], and deoxyribonucleic acid (DNA) damage induced by oxidative stress is considered to be a key mechanism of PM_2.5_-mediated genotoxicity [6]. PM_2.5_ adsorbs hazardous constituents such as transition metals and organic compounds, which generate reactive oxygen species (ROS) in organisms [7,8]. ROS produced by oxidative stress can over oxidize cellular biomolecules, and DNA is considered to be an important target for ROS [9]. ROS can result in increased levels of strand-breaking effects and DNA base oxidations [10,11]. Compared to DNA single-strand breaks (SSBs), cells suffer fatal and highly cytotoxic outcomes from double-strand breaks (DSBs) along the resulting spectrum of DNA lesions [12]. When endogenous or exogenous DSBs occur, the phosphorylated histone proteins H2AX (γ-H2AX) gather at the damage sites extensively to form foci and trigger the repair response [13]. If DNA damage is not repaired properly prior to DNA replication, the potentially malignant consequences are illustrated by the fact that cells will present DNA damage responses (DDR) such as DNA mutation, replication errors, genomic instability, and even cell death [14]. Base excision repair (BER) is an important DNA repair pathway, which is responsible for the oxidized bases, apurinic/apyrimidinic (AP) sites, and DNA strand breaks in human cells [15].

PM_2.5_ is a heterogeneous mixture of chemical species with complexity and variability. The specific PM_2.5_ composition changes with the season, source, and geography [16]. Increasing evidence has shown that the source of PM_2.5_ is an important risk factor for the impact on toxic effects [17,18]. Studies have suggested that the genotoxicity of PM_2.5_ organic extracts from European cities changes depending upon the source of PM_2.5_ [19]. The PM_2.5_ in Beijing is characterized by seasonal pollution emissions, due in part to heating during Beijing winters. Soot emissions from industrial boilers and heating boilers have increased significantly, creating a unique air pollutants of Beijing [20,21]. Although numerous studies have reported the genotoxicity induced by PM_2.5_, the detailed genotoxicity data of Beijing PM_2.5_ are currently limited and warrant further detailed investigations. Therefore, PM_2.5_ from Beijing in winter was used as a model sample in this study. This study focused on the genotoxicity of PM_2.5_ in vitro and the associated oxidative stress and DNA repair genes. The results indicated that PM_2.5_ from Beijing strongly induced DNA strand breaks and oxidative DNA damage in the human bronchial epithelial cell line 16HBE, which is potentially caused by oxidative stress and the influence of DNA repair capacity.

## 2. Materials and Methods

### 2.1. Collection of PM_2.5_ Samples

PM_2.5_ samples were collected at the Beijing University of Technology, Beijing, China, from 1 January to 31 January 2017. PM_2.5_ was continuously sampled by high-volume samplers (Tianhong, China) at a flow rate of 1 m^3^/min and collected on quartz filters (47 mm, 2 µm, Whatman), according to our previous study [22]. The quartz fibers were ultrasonicated for four cycles of 5 min each to prevent overheating. Then the sonicated eluate was filtered using 6 layers of gauze and dried in a vacuum frozen desiccator. After weighing, the pellets of PM_2.5_ were resuspended in ultrapure water to prepare a stock solution of 4 mg/mL.

### 2.2. Cell Culture and Treatment

In this study, human bronchial epithelial cells 16HBE were obtained from Shanghai SXBIO Biotechnology (SXBIO Biotechnology, Shanghai, China). 16HBE were grown in Roswell Park Memorial Institute (RPMI) 1640 medium (Gibco BRL, New York, NY, USA) supplemented with 10% fetal bovine serum and 1% anti-penicillin/streptomycin (100 U/mL), cultured at 37 °C with 5% CO_2_. For PM_2.5_ exposure, the particle suspension was sonicated for 2 min in ice-water bath and then mixed to the culture medium to reach the concentration we need. For cell viability assay, 16HBE were treated with PM_2.5_ at final concentrations of 25, 50, 100, 200, and 400 μg/mL for 24 h. For ROS determination, 202.5 μg/mL PM_2.5_ was applied to cells for 0.5, 1, 2, 3, and 4 h. For other experiments, PM_2.5_ at low concentrations of 67.5 μg/mL for 30% inhibitory concentration (IC30), medium concentration of 116.9 μg/mL for 50% inhibitory concentration (IC50), and high concentration of 202.5 μg/mL for 70% inhibitory concentration (IC70) following CCK-8 assay were added to the cells for 24 h.

### 2.3. Cell Viability Assay

Cell viability assay were performed following manufacturer instructions using the Cell Counting Kit-8 (CCK-8) (Dojindo Molecular Technologies, Inc., Kumamoto, Japan). After exposure, 10 μL of CCK-8 was added to each well, which was incubated at 37 °C for 2 h. The absorbance was read at 450 nm by an EnSpire^®^ Multimode Plate Reader (PerkinElmer Inc., Waltham, MA, USA). The viability of the untreated control cells was set as 100%.

### 2.4. Lactate Dehydrogenase (LDH) Release

To measure the effect of PM_2.5_ on cell membrane damage, the level of LDH released from 16HBE cells was measured. After PM_2.5_ exposure, the level of LDH from the supernatant of each experimental sample or control sample was measured according to the manufacturer’s protocols of the LDH assay kit (Nanjing Jiancheng Biochemistry, Nanjing, China). The absorbance was read at the wavelength of 490 nm by an EnSpire^®^ Multimode Plate Reader (PerkinElmer Inc., Waltham, MA, USA).

### 2.5. Comet Assay

Comet assay was performed following the instructions of the comet assay kit (KeyGEN BioTECH, Jiangsu, China) to make 3 layers of gel on the slides. The first layer was 0.5% normal melting point agarose; the second layer was 0.7% low melting agarose, and the third layer was 0.7% low melting agarose. The slides were lysed in lysis buffer for 2 h, and then immersed in an alkaline electrophoresis solution to untwist for 30 min. After single-cell electrophoresis, the slides were stained with propidium iodide (PI) for 10 min, and images were obtained with a laser confocal microscope (Leica, Solms, Germany). Tail DNA, tail length and olive tail moment (OTM) were analyzed through Comet Assay Software Project (CASP) [23].

### 2.6. Western Blot

The cells were lysed with radio immunoprecipitation assay (RIPA) lysate buffer (Cell Signal Technology, Danvers, MA, USA) to obtain the total protein. After centrifugation at 13,000× *g* for 10 min, the protein concentration was measured using the bicinchoninic acid (BCA) protein quantification kit. Protein was separated on a 10% or 12% SDS-PAGE gel and then blotted onto a PVDF membrane. After blocking with 5% BSA for 2 h, the membrane was incubated overnight at 4 °C with the following primary antibodies: γ-H2AX antibody (abcam, Cambridge, England), 8-oxoguanine DNA glycosylase (OGG1) antibody (Proteintech, Wuhan, China), poly (ADP-ribose) polymerase-1 (PARP1) antibody (Proteintech, Wuhan, China), X-ray repair cross-complementing gene 1 (XRCC1) antibody (Proteintech, Wuhan, China), HO-1 antibody (Proteintech, Wuhan, China), glyceraldehyde-3-phosphate dehydrogenase (GAPDH) antibody (Bioworld, Nanjing, China), and β-actin antibody (abcam, Cambridge, England). The secondary antibody was RDye800^®^ conjugated goat anti-rabbit IgG (KPL, Gaithersburg, MD, USA) and RDye800^®^ conjugated goat anti-mouse IgG (KPL, Gaithersburg, MD, USA), incubated at room temperature for 1 h. Finally, the bands were obtained using an Odyssey infrared imaging system (LI-COR Biosciences, Lincoln, NE, USA).

### 2.7. Immunofluorescence

16HBE cells were washed with phosphate buffered saline (PBS), fixed with 4% paraformaldehyde for 15 min, and permeabilized with 0.3% Triton X-100 solution for 15 min at room temperature. Then, the cells were blocked with 10% donkey serum for half an hour at room temperature to block nonspecific binding. The cells were incubated with γ-H2AX antibody (abcam, Cambridge, England) for the primary antibody overnight at 4 °C and DyLight 488 donkey anti-mouse IgG (Earthox, Millbrae, CA, USA) for the secondary antibody 1 h at room temperature. Then, the nuclei were stained with Hoechst 33258 at room temperature for 10 min in the dark. Images were obtained through a laser confocal microscope (Leica, Solms, Germany).

### 2.8. Enzyme-Linked Immuno Sorbent Assay (ELISA)

After extracting the total protein of the cells with the RIPA lysate, the total protein concentration was measured using a BCA protein quantification kit (Solarbio, Bejing, China). The level of 8-OH-dG content assay was performed using the human 8-OHdG ELISA kit (Meimian, Jiangsu, China) following the manufacturer’s instructions. The value of optical density (OD) was obtained at 450 nm using an EnSpire^®^ Multimode Plate Readers (PerkinElmer Inc., Waltham, MA, USA).

### 2.9. Micronucleus (MN) Assay

The cells were fixed with 4% paraformaldehyde for 15 min at room temperature and then stained with Hoechst 33258 for 20 min. At each step, the cells were washed three times with PBS. In each group, 1000 cells were randomly observed using a fluorescence microscope (Zeiss, Oberkochen, Germany), and the number of cells containing MN was counted to calculate the MN rate.

### 2.10. ROS Assay

After PM_2.5_ exposure, 10 μM 2′,7′-dichlorohydrofluorescein diacetate (DCFH-DA) probe was added to each well, incubated at 37 °C for 30 min. Then each well was washed three times with PBS, and the fluorescence intensity was detected by an EnSpire^®^ Multimode Plate Readers (PerkinElmer Inc., Waltham, MA, USA) at an excitation wavelength of 488 nm and an emission wavelength of 525 nm.

### 2.11. Measurement of Reduced Glutathione (GSH) and Malondialdehyde (MDA)

After PM_2.5_ exposure, the levels of GSH and MDA from 16HBE cells were measured using the corresponding kits (Beyotime Biotechnology, Shanghai, China) following the manufacturer’s instructions, and the data were obtained by using an EnSpire^®^ Multimode Plate Reader (PerkinElmer Inc., Waltham, MA, USA).

### 2.12. Statistical Analysis

Data were expressed as mean ± standard deviation (SD) for at least three independent experiments. Statistical analysis was performed by using GraphPad Prism 6 software. Statistical differences were compared between the experimental group and the control group by the homogeneity test of variance and the independent sample t test. The statistical significance was defined as ** *p* < 0.01, * *p* < 0.05.

## 3. Results

### 3.1. PM_2.5_ Induced Cytotoxicity

**Figure 1:**
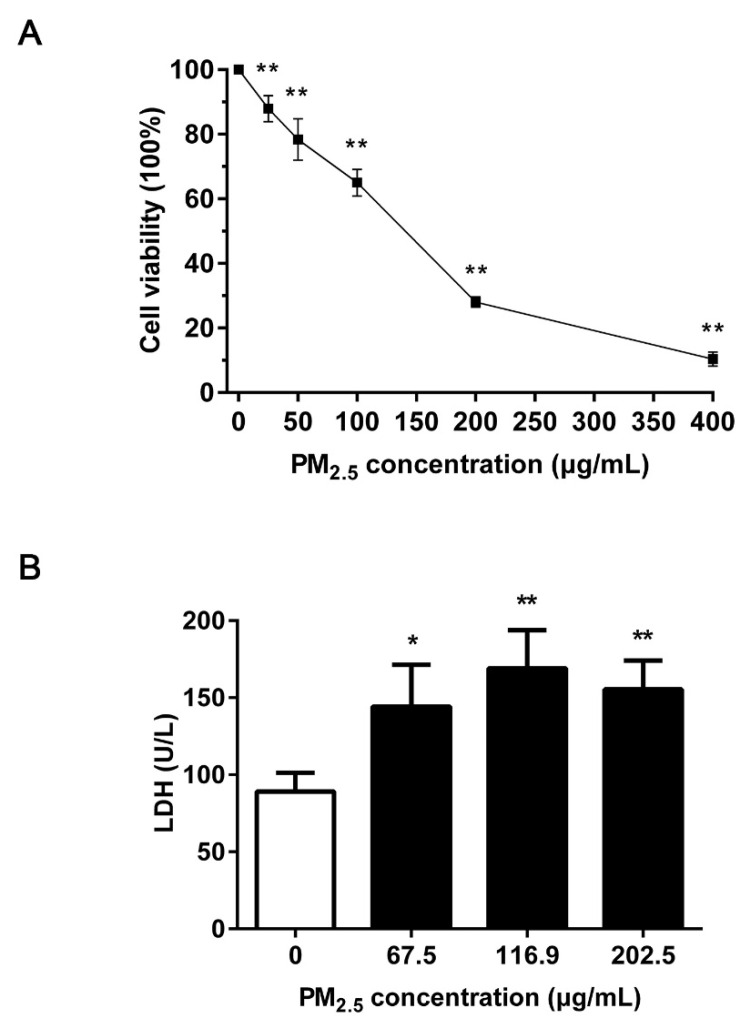
Effects of PM_2.5_ on cell cytotoxicity. (**A**) Analysis of cell viability. 16HBE were treated with 0–400 μg/mL of PM_2.5_ for 24 h. (**B**) Analysis of the level of lactate dehydrogenase (LDH). 16HBE exposed to 67.5, 116.9, 202.5 μg/mL PM_2.5_ for 24 h. Data are shown as mean ± standard deviation (SD) (*n* = 5). * *p* < 0.05 compared with control. ** *p* < 0.01 compared with control.

After exposure to PM_2.5_ for 24 h, the cell viability was tested by CCK-8 methods. When the concentration of PM_2.5_ reached 25 μg/mL, the cell viability of 16HBE was significantly reduced compared with the control group (*p* < 0.01), and the cell viability decreased sharply with dose dependent on PM_2.5_ (Figure 1A). After calculation, IC30 = 67.5 μg/mL, IC50 = 116.9 μg/mL, and IC70 = 202.5 μg/mL, representing the low, medium and high doses of PM_2.5_, respectively.

The level of LDH in the supernatant represents the damage to the cell membrane. A significant increase in the level of LDH was observed in 16HBE cells after exposure to PM_2.5_ (Figure 1B) (*p* < 0.05 or *p* < 0.01). These suggested the cytotoxicity of PM_2.5_ on 16HBE.

### 3.2. PM_2.5_ Induced 16HBE Oxidative Stress

**Figure 2:**
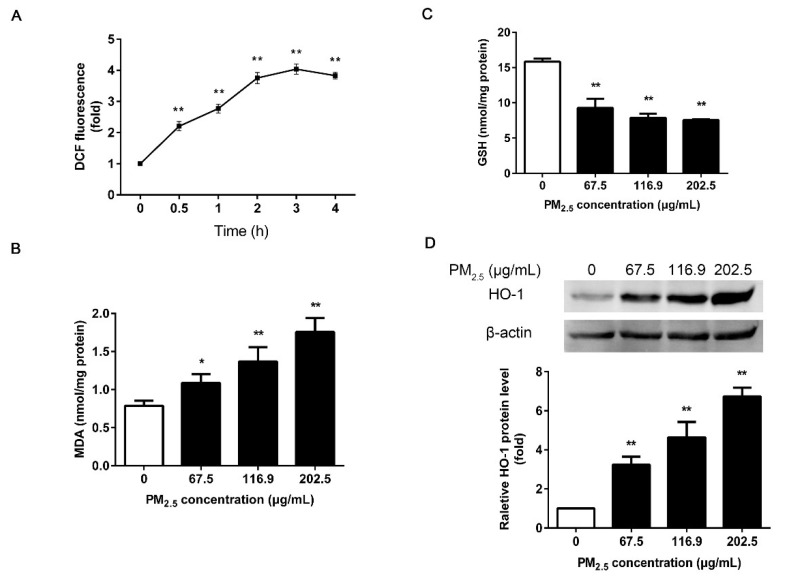
Effects of PM_2.5_ on oxidative stress. (**A**) Detection of ROS production in 16HBE by DCFH-DA probe. 16HBE were exposed to 202.5 μg/mL PM_2.5_ for 0–4 h. (**B**) Analysis of malondialdehyde (MDA). 16HBE exposed to 67.5, 116.9, 202.5 μg/mL PM_2.5_ for 24 h. (**C**) Analysis of glutathione (GSH). (**D**) Analysis of HO-1 by western blot. β-actin was used as a loading control. Data are shown as mean ± SD (*n* = 3). * *p* < 0.05 compared with control. ** *p* < 0.01 compared with control.

To investigate whether PM_2.5_ induces oxidative stress in 16HBE, we first measured the ROS production in the cells by DCFH-DA probe. Compared with the control group, the fluorescence intensity increased by about 2 times after 0.5 h of exposure (Figure 2A), indicating that the ROS generation in a very short time. The ROS increased in a time-dependent manner, and the fluorescence intensities gradually became stable at 3 h (*p* < 0.01).

MDA and GSH are typical markers of oxidative stress. MDA represents the level of lipid peroxidation products, and GSH represents the antioxidant enzyme system. Quantitative analysis indicated that PM_2.5_ caused a significant increase in MDA in a dose-dependent manner (Figure 2B), while GSH was greatly reduced after exposure (Figure 2C) (*p* < 0.05 or *p* < 0.01).

Next, we examined the expression level of cellular heme oxygenase (HO-1), one of the oxidative stress response enzymes. We confirmed the PM_2.5_-driven up-regulation of HO-1 protein in 16HBE through western blot analysis (*p* < 0.01) (Figure 2D). These results suggested that PM_2.5_ induced severe oxidative stress in 16HBE.

### 3.3. PM_2.5_ Caused 16HBE DNA Strand Breakages

**Figure 3:**
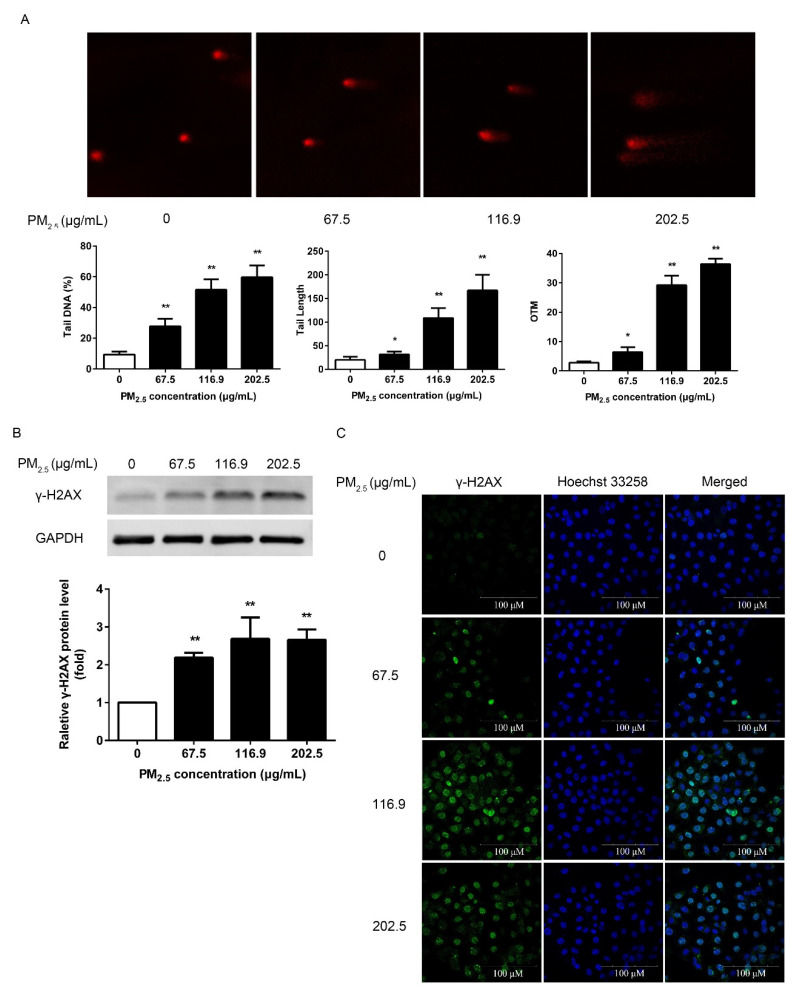
Effect of PM_2.5_ on deoxyribonucleic acid (DNA) strand breaks. 16HBE were exposed to 67.5, 116.9, 202.5 μg/mL PM_2.5_ for 24 h. (**A**) The comet analysis of PM_2.5_ on DNA fragmentation. (**B**) The analysis of PM_2.5_ on the expression of γ-H2AX by western blot. Glyceraldehyde-3-phosphate dehydrogenase (GAPDH) was used as a loading control. (**C**) Immunofluorescence of γ-H2AX in 16HBE. Data are shown as mean ± SD (*n* = 3). * *p* < 0.05 compared with control. ** *p* < 0.01 compared with control.

DNA strand breaks are an important indicator for evaluating the biological characteristics of cells. The comet assay can very well illuminate SSBs or DSBs. The results showed that compared with the control, a progressive increase in DNA tail (%) was observed following PM_2.5_ exposure (Figure 3A). Moreover, both tail length and OTM elevated significantly in a dose-dependent manner (*p* < 0.05 or *p* < 0.01).

The formation of γ-H2AX is considered to be a biomarker of DSBs. In this study, we used western blot and immunofluorescence to confirm the formation of γ-H2AX. Western blot results showed that compared with the control group; the level of γ-H2AX was significantly increased with the concentration of PM_2.5_ (*p* < 0.01) (Figure 3B). The results of immunofluorescence also confirmed the results of western blot. Compared with the control group, PM_2.5_ markedly enhanced the number of γ-H2AX foci and fluorescence intensity (Figure 3C).

### 3.4. PM_2.5_ Caused 16HBE Oxidative DNA Damage

**Figure 4:**
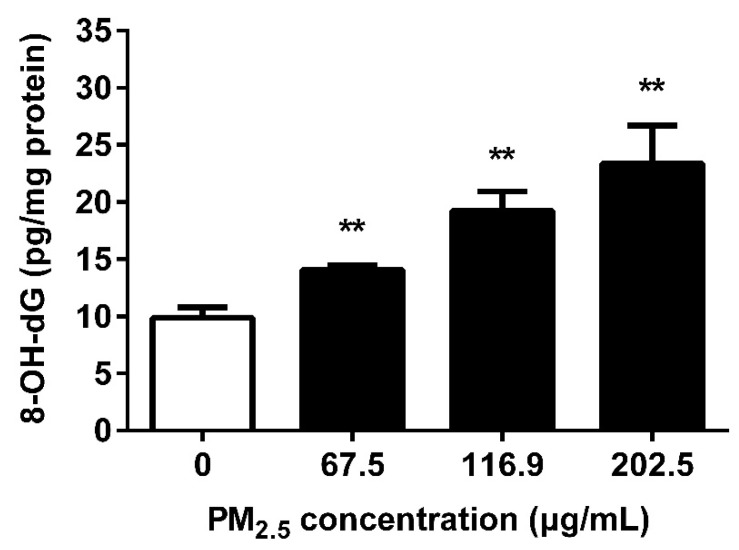
Effect of PM_2.5_ on oxidative DNA damage. 16HBE were exposed to 67.5, 116.9, 202.5 μg/mL PM_2.5_ for 24 h. The level of 8-OH-dG in 16HBE was detected by enzyme-linked immuno sorbent assay (ELISA) Data are shown as mean ± SD (*n* = 3). ** *p* < 0.01 compared with control.

To assess the ability of oxidative DNA damage, 16HBE were treated with PM_2.5_ for 24 h and the level of 8-OH-dG was determined by ELISA. The results showed an elevation of 8-OH-dG in 16HBE upon exposure to PM_2.5_ starting at 67.5 μg/mL and with a peak at 202.5 μg/mL (*p* < 0.01) (Figure 4).

### 3.5. PM_2.5_ Caused 16HBE Chromatin Damage

**Figure 5:**
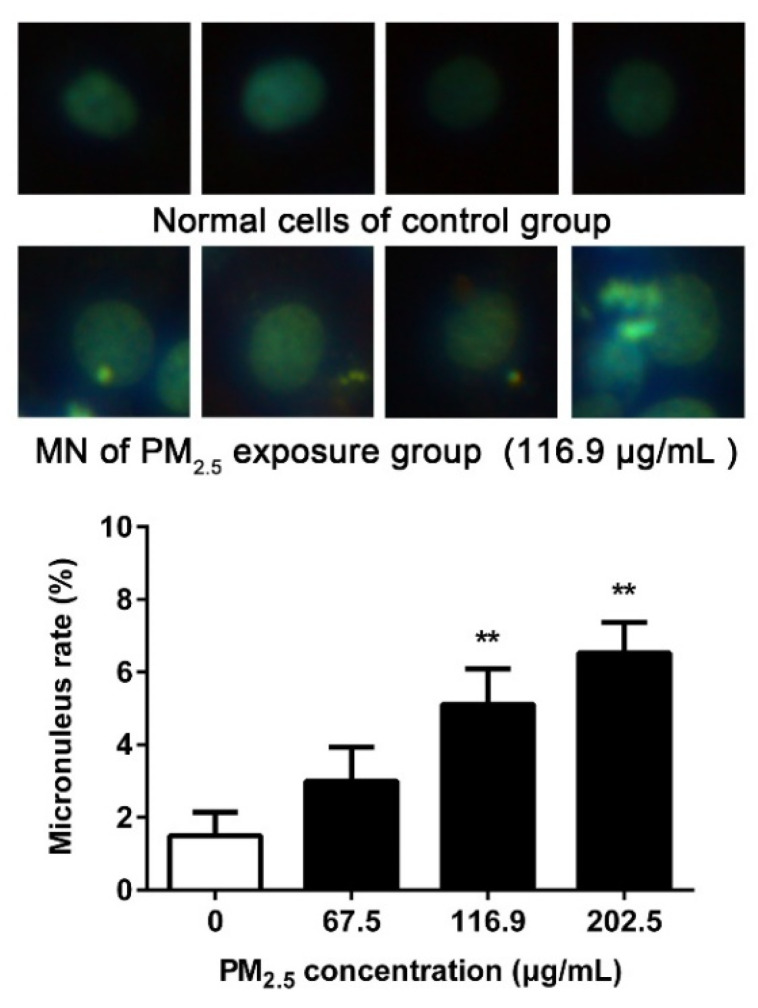
Effect of PM_2.5_ on MN. 16HBE were exposed to 67.5, 116.9, 202.5 μg/mL PM_2.5_ for 24 h. Data are shown as mean ± SD (*n* = 3). ** *p* < 0.01 compared with control.

MN assay is used to assess DNA damage at the chromosomal level. Compared with the control group, the MN rate of 16HBE increased statistically after the dose reached 116.9 μg/mL (Figure 5), and the rate enhanced in a dose-dependent manner by the exposure of PM_2.5_ (*p* < 0.01).

### 3.6. Effects of PM_2.5_ on DNA Repair Genes

**Figure 6:**
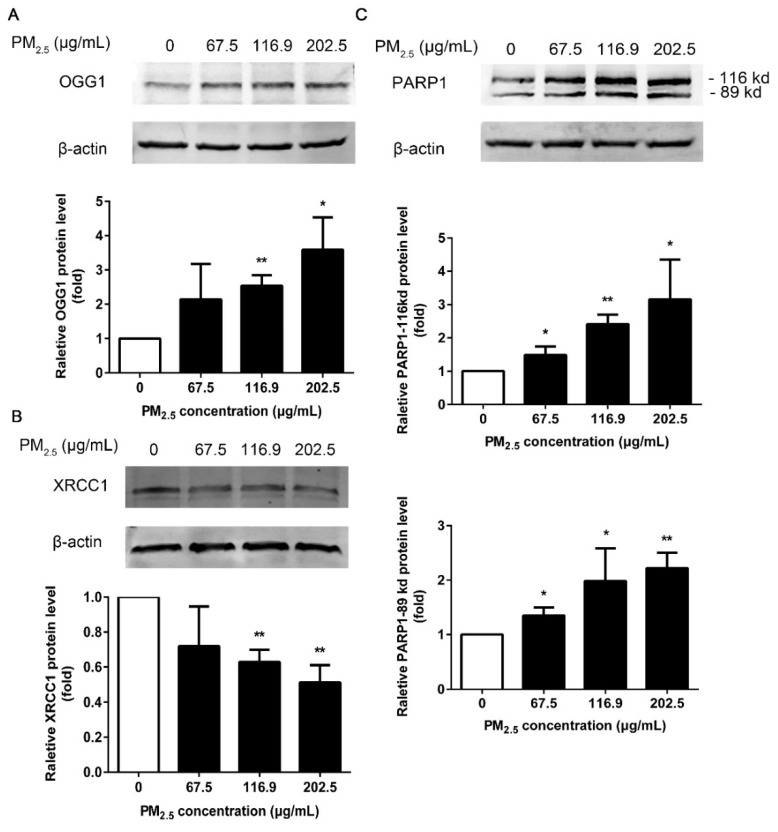
Effect of PM_2.5_ on DNA repair genes. Western blot of 16HBE exposed to 67.5, 116.9, 202.5 μg/mL PM_2.5_ for 24 h. (**A**) Expression of OGG1. (**B**) Expression of XRCC1. (**C**) Expression of PARP1. β-actin was used as a loading control. Data are shown as mean ± SD (*n* = 3). * *p* < 0.05 compared with control. ** *p* < 0.01 compared with control.

In this study, we demonstrated PM_2.5_-influenced repair gene expression, such as OGG1, XRCC1, and PARP1. There was no statistical increase in OGG1 expression at a concentration of 67.5 μg/mL compared to the control group (Figure 6A). The increase in OGG1 expression was significant at concentrations of 116.9 and 202.5 μg/mL in 16HBE cells after exposure. While PM_2.5_ significantly inhibited the expression of XRCC1 at concentrations of 116.9 and 202.5 μg/mL compared to the control (Figure 6B). In contrast, a statistically significant enhancement of both 116 and 89 kDa of PARP1 was reported in 16HBE cells, irrespective of exposure doses (Figure 6C) (*p* < 0.05 or *p* < 0.01).

## 4. Discussion

According to large and growing evidence of epidemiological and experimental research, exposure to PM_2.5_ is associated with morbidity and mortality from respiratory diseases including lung cancer [23]. PM_2.5_ has been well proven for the induction of cell genotoxicity. However, the degree of genotoxicity of PM_2.5_ varies depending on its source and chemical components. There is still a lack of detailed data on the genotoxicity of PM_2.5_ in Beijing. Our previous studies found that these PM_2.5_ samples from Beijing adsorbed 20 metals and 15 priority US EPA polycyclic aromatic hydrocarbons (PAHs), which is suggested to be the key toxic substances and mutagenic substances of PM_2.5_ [24]. Among these, chromium, arsenic, cadmium, nickel, and lead are carcinogenic metals, while benzo(k)fluoranthene, benzo(a)pyrene benzo(a)anthracene, dibenzo(a)anthracene, and benzo(b)fluoranthene have been classified as carcinogens by the International Cancer Research Agency (IARC). Therefore, it is important to study the genotoxicity of PM_2.5_ from Beijing. In this study, we found that PM_2.5_ from Beijing resulted in DNA strand breaks, elevated levels of 8-OHdG and chromosomal damage in human bronchial epithelial cells 16HBE, which might be mediated partially by oxidative stress and the influence of DNA repair capacity.

First, exposure to PM_2.5_ can cause significant concentration-dependent cytotoxicity in 16HBE, resulting in decreased cell viability and cell membrane destruction. According to the results of acute cytotoxicity assay, the following genotoxic assays were conducted with low, medium, and high doses of PM_2.5_ (IC30, IC50, and IC70). ROS-mediated oxidative damage is considered to be one of the critical mechanisms of DNA damage. A recent study showed PM_2.5_ induced DSBs in HCEC cells through the accumulation of ROS and oxidative stress [25]. Diesel exhaust particles increased the markers of oxidative stress and induced DNA damage in the mouse heart [26]. In addition, previous studies revealed that environmental pollutants caused DNA damage in H9c2 cells by promoting the accumulation of intracellular ROS and superoxide anions [27]. We have measured ROS, oxidative stress markers (MDA and GSH), and HO-1 to show that PM_2.5_ enhanced ROS generation and eliminated the homeostasis of oxidative stress, causing a severe oxidative tendency in cells. These findings are consistent with the report that PM_2.5_ (0–200 µg/mL) from Shanghai induced oxidative stress in human keratinocytes and human melanocytes, which was manifested as an increase in MDA and HO-1 [28]. MDA is a product of lipid peroxidation, while GSH acts as a ROS scavenger, so it reflects the severity of the cells attacked by ROS. HO-1 is also a typical inducible stress response enzyme that plays an essential role in regulating the cellular antioxidative defense [29]. Based on reports, the chemical composition of PM_2.5_ plays a pivotal role in the contribution of PM_2.5_-induced oxidative stress (Jin et al. 2019). The adsorptive pollutants of soluble metals and PAHs may be closely related to the increased ROS. We have reported that transition metals and PAHs in our Beijing PM_2.5_ compositions [24]. According to our report, the Beijing PM_2.5_ adsorbs soluble transition metals on its surface, including iron, copper, chromium, and vanadium, which can generate ROS through Fenton type reactions. The bioavailable transition metals on the particle surfaces have been separately demonstrated to contribute to the generation of ROS in biological systems [6,30]. Furthermore, there was a strong and direct correlation between ROS generation and PAHs content [31]. Research has shown that PAHs were the major redox-active components on PM_2.5_ [32]. The metabolism of PAHs can cause a generation of hydrogen peroxide and superoxide anions [33]. Jin et al. quantified the contribution of metals and PAHs to the in vitro oxidative stress caused by PM_2.5_ from Beijing in winter. Metals and PAHs explained 38% on average of ROS induced by PM_2.5_ in Beijing. PAHs contributed approximately twice the share of the PM_2.5_ mixture effects as metals [34].

The overproduction of ROS may attack and destroy various cellular macromolecules including DNA, proteins, peptides, and lipids [35]. It is well known that ROS are capable of interacting with DNA molecules to induce DNA strand breaks and cause oxidative DNA damage [36]. Among oxidative DNA lesions, 8-OH-dG is the predominant and most abundant oxidative product induced by oxygen free radicals formed in the nuclear DNA or mitochondrial DNA and is a suitable marker for assessing the individual endogenous oxidative DNA damage [37]. PM_2.5_ exposure has been reported to cause elevated levels of 8-OH-dG in RPMI 1788 and A549 cells [38]. Enhancement of 8-OH-dG and ROS generation in 16HBE cells showed that DNA was damaged by PM_2.5_-driven excessive ROS. Moreover, ROS is a potent inducer to make DNA strand break by directly oxidizing the bases of DNA or by covalent bonding of MDA to DNA [39]. Gao et al. showed that the ROS produced by PM_2.5_ from Guangzhou greatly contributed to the genotoxicity of PM_2.5_ [25]. PM collected from a small village of Danish can have extensive contribution of 8-OH-dG generation in A549 cells. It generated ROS and DNA damage in a dose-dependent manner [40]. Our results are consistent with these researches. The comet assay is a sensitive and reliable method for DNA damage detection [23]. Our study showed that PM_2.5_ was capable of elevating the important indicators of the comet assay, confirming that PM_2.5_ significantly damaged the cellular DNA, and DNA strand breaks occurred. The comet assay showed that PM collected from Korea increased DNA damages in Chinese hamster ovary cells and human normal bronchial cells, and this damage was partly due to oxidative damage to DNA [41], which supports our results. Zou et al. detected the genotoxicity of PM_2.5_ (0–200 µg/mL) from Shanghai by comet assay. According to the results of OTM, it is posited that Beijing PM_2.5_ has more significant toxicity [42]. Since DSBs represent the most severe DNA damage caused by oxidative stress, we further confirmed the DSBs levels after PM_2.5_ exposure. γ-H2AX is considered to be used as a biomarker of DSBs. The 139th serine of H2AX is extensively phosphorylated within 1–3 min to form foci at damage sites after endogenous or exogenous DSBs [43]. These foci represent the DSBs in a 1:1 manner and the number of foci increases linearly with the severity of the damage. Our study reported the formation of γ-H2AX after PM_2.5_ exposure, indicating that PM_2.5_ induced DSBs in 16HBE. Our result is consistent with the result of Yang et al., that PM_2.5_ from six different cities in China including Beijing induced γ-H2AX formation in BEAS-2B cells [44]. PM_2.5_ from rural and urban areas of Lebanon also showed the induced effect of γ-H2AX [45]. DSBs are known to represent chromosomal lesions, so we detected chromosomal stability by MN assay after PM_2.5_ exposure. The formation of MN is caused by an abnormal break of chromosomes during the cell division stage and remains in the cytoplasm, which represents DNA damage at the chromosomal level [46]. MN also reflects the capacity to repair DNA damage of cells. The more severe the chromosomal damage, the worse the DNA repair ability. Bocchi et al. reported that PM_2.5_ from Bologna, a county town of Emilia-Romagna in the north of Italy, induced chromosome breakage in A549 cells [47], which is consistent with the results we observed in 16HBE. Our available data suggest that PM_2.5_ induced DNA damage in 16HBE cells in a dose-dependent manner and that even exposure to low concentrations of PM_2.5_ may result in significant cellular DNA damage. This may lead to the accumulation of mutations in cells, promoting the development of cancer.

DNA repair genes play a crucial role in regulating the genotoxic effects of environmental carcinogens. If DNA damage fails to be repaired before replication, accumulation of DNA damage, such as DNA strand breaks and 8-OH-dG formation, can lead to heritable DNA mutations or abnormal gene expression, leading to potential genetic disease. BER is an important DNA repair pathway in human cells responsible for repairing the vast majority of small adducts, such as DNA oxidation modifications [48]. Therefore, it is reasonable to investigate the effect of PM_2.5_ on the BER pathway. OGG1 is one of the essential enzymes in BER, which can excise 8-OH-dG [49]. XRCC1 acts as a scaffold protein to recruit and organize the sets of enzymes required in the multi-step repair process, playing a central role in the BER [50]. PARP1 can be rapidly recruited to DNA damage sites and promotes DNA damage repair in BER through multiple functions [51]. The role of PARP1 in DNA strand breaks has been well characterized [52]. Mehta et al. reported that PM inhibited nucleotide excision repair (NER) of cells and enhanced both DNA replication errors and carcinogen-induced mutagenesis [53]. Li et al. found that PM_2.5_ from Taiyuan considerably activated BER repair gene OGG1 and inhibited XRCC1 expression in the hearts of rats [54]. Our results are consistent with previously reported study results, in which PM_2.5_ elevated OGG1 and inhibited XRCC1 expression. The 89 kDa of PARP1 originates from the early phase of apoptosis, whereby PARP1 is cleaved by caspases into an 89 kDa and a 24 kDa fragment and lost repair activity [55]. Notably, although PARP1 showed an increasing trend in the 116 kDa protein, the level of 89 kDa fragment was also enhanced, indicating that PARP1 gradually lost enzymatic activity. These results revealed that the DNA repairing process is influenced by PM_2.5_ exposure in 16HBE.

## 5. Conclusions

(1) PM_2.5_ from Beijing markedly induced cytotoxicity.
(2) PM_2.5_ significantly promoted oxidative stress accompanied by the increases of ROS, MDA and HO-1, while the level of GSH was decreased.
(3) PM_2.5_ caused DNA damage effects involved in DNA strand breaks, 8-OHdG formation and MN formation, indicating that the DNA of 16HBE was indeed damaged by ROS.
(4) PM_2.5_ significantly influenced DNA repair genes. OGG1 was enhanced and XRCC1 was decreased. The influence of repair ability will lead to the occurrence of DNA damage, which may be one of the mediating factors of PM_2.5_ genotoxicity.

Our work provided an additional evidence for the genotoxicity effects of PM_2.5_ from Beijing in winter, and the possible mechanisms involved.
